# Supplementary material for: Complexome profiling of the Chlamydomonas psb28 mutant reveals TEF5 as an early PSII assembly factor
Source: Plant Cell. 2025 Mar 17;37(6):koaf055. doi: 10.1093/plcell/koaf055 (PMC12164590; doi:10.1093/plcell/koaf055)
Supplement: koaf055_Supplementary_Data [file koaf055_supplementary_data.zip › psb28_interactive.zip!/plots/intensity/qualityPlots/peptides/petD_Cre-1.g2717021.t1.p1.html]

### Cre-1.g2717021.t1.p1

Trivial name: petD  
SeqCoverage [median]: 13.80  
Mass: 17.44 kDa  
  
 

| Sequence | MissedCleavage | Mass [Da] | wtIntensity | muIntensity | Score |
| --- | --- | --- | --- | --- | --- |
| FQNPYR | 0 | 823.40 | 75922.37 | 140003.69 | 156.22 |
| KPDLSDPVLK | 1 | 1110.63 | 203519.52 | 192042.44 | 225.67 |
| KPDLSDPVLKAK | 2 | 1309.76 | 8554.16 | 25081.20 | 156.00 |
| SVTKKPDLSDPVLK | 2 | 1525.87 | 461048.33 | 819528.69 | 172.85 |
| SVTKKPDLSDPVLKAK | 3 | 1725.00 | 7408.97 | 104500.91 | 133.86 |
|  |  |  | 756453.4 | 1281156.9 |  |
